# Medical and Surgical Report of the Presbyterian Hospital, New York

**Published:** 1902-12

**Authors:** 


MEDICAL AND SURGICAL REPORT OF THE PRESBYTERIAN HOSPITAL, NEW YORK. Volume V. January, 1902. Edited by DRS. ANDREW J. MCCOSH and W. GILMAN THOMPSON.

Volume V. of the Presbyterian Hospital (New York) Reports, edited by Drs. Andrew McCosh and W. Gilman Thompson is a credit to the hospital and its medical staff. Twenty-four papers are contributed, each one interesting as well as instructive, and serve as an index of the work done at this popular hospital. The general excellence of the papers is noteworthy, many of them being accompanied by carefully prepared illustrations. Among the more important of the contributions may be mentioned the following:

Tumor of the brain by M. Allen Starr and A. J. McCosh; Acute pancreatitis with the report of three cases, by George Woolsey; Two papers relating to ureter catheterisation, by F. Tilden Brown; A report of twenty-two cases of abscess of the brain, John W. Coe and Burton B. Lee; Wandering kidney and the results of operation, Clarence A. McWilliams; The pathological condition found at 1,000 autopsies, John S. Thatcher; A case of dislocation of the spine resulting in crush of the sixth cervical segment—irregularity in the distribution of the paralysis, A. J. McCosh and George A. Tuttle; three blood analyses, Fred P. Solley; Report of a case of tumor of the cauda equina, with successful operation for removal—recurrence—death, M. A. Starr and A. J. McCosh; A case of gangrene of the small intestine, due to hematoma in mesentery, as a result of contusion of abdomen, Andew J. McCosh; A rare case of intestinal obstruction, E. Eliot, Jr.; Unusual metrorrhagia, Sidney S. Graber; Cases of multiple exostosis, Forbes Hawkes; A case of epidemic cerebrospinal meningitis, with an unusual skin lesion, Linsly R. Williams; Calculus of the right ureter, loss of function in the right kidney, with compensating hyper-secretion of the left, removal of calculus by the transperitoneal route, recovery, Forbes Hawkes; A case of subcutaneous rupture of the trachea, with remarks on forty-three similar injuries, Alfred A. Osgood; Sarcoma of external popliteal nerve, excision of three and one-half inches of nerve, union of ends by plastic operation, Andrew J. McCosh; An extension splint for Colles's fracture, Reginald H. Jackson; Fractures and dislocations in the emergency ward of the Presbyterian Hospital for the two years from October 1, 1899, to September 30, 1901, Alfred T. Osgood.

In appearance the volume is similar to the preceding ones and serves as a good example for other hospitals to follow.

